# On Identifying and Mitigating Bias in Inferred Measurements for Solar Vector Magnetic-Field Data

**DOI:** 10.1007/s11207-022-02039-9

**Published:** 2022-09-14

**Authors:** K. D. Leka, Eric L. Wagner, Ana Belén Griñón-Marín, Véronique Bommier, Richard E. L. Higgins

**Affiliations:** 1 NorthWest Research Associates, Boulder, CO USA; 2 Institute for Space-Earth Environmental Research, Nagoya University, Nagoya, Aichi Japan; 3 W.W. Hansen Experimental Physics Laboratory, Stanford University, Stanford, CA USA; 4 Institute of Theoretical Astrophysics, University of Oslo, Blindern, Oslo, Norway; 5 Rosseland Centre for Solar Physics, University of Oslo, Blindern, Oslo, Norway; 6 LESIA, Observatoire de Paris, Université PSL, Sorbonne Université, Université Paris Cité, CNRS, Meudon, France; 7 University of Michigan, Ann Arbor, MI USA

**Keywords:** Instrumental effects, Magnetic fields, photosphere, Polarization, optical

## Abstract

The problem of bias, meaning over- or under-estimation, of the component perpendicular to the line-of-sight [$B_{\perp }$] in vector magnetic-field maps is discussed. Previous works on this topic have illustrated that the problem exists; here we perform novel investigations to quantify the bias, fully understand its source(s), and provide mitigation strategies. First, we develop quantitative metrics to measure the $B_{\perp }$ bias and quantify the effect in both local (physical) and native image-plane components. Second, we test and evaluate different options available to inversions and different data sources, to systematically characterize the impacts of these choices, including explicitly accounting for the magnetic fill fraction [$f\!\!f$]. Third, we deploy a simple model to test how noise and different models of the bias may manifest. From these three investigations we find that while the bias is dominantly present in under-resolved structures, it is also present in strong-field, pixel-filling structures. Noise in the spectropolarimetric data can exacerbate the problem, but it is not the primary cause of the bias. We show that fitting $f\!\!f$ explicitly provides significant mitigation, but that other considerations such as the choice of $\chi ^{2}$-weights and optimization algorithms can impact the results as well. Finally, we demonstrate a straightforward “quick fix” that can be applied post facto but prior to solving the $180^{\circ}$ ambiguity in $B_{\perp }$, and which may be useful when global-scale structures are, e.g., used for model boundary input. The conclusions of this work support the deployment of inversion codes that explicitly fit $f\!\!f$ or, as with the new SyntHIA neural-net, that are trained on data that did so.

## Introduction

It is a challenging problem to infer the magnetic-field strength and direction in the solar photosphere, as it threads a dynamic plasma (see del Toro Iniesta and Ruiz Cobo, 2016, and references therein). The assumptions of a Milne–Eddington (ME) atmosphere provide a good estimate of the average strength and direction across the layers where magnetically sensitive photospheric spectral lines are formed (Westendorp Plaza et al., 1998), especially in structures where a single pixel-filling magnetic component is present or at least dominant. Under-resolved multiple atmospheres (whether all magnetized or not) contributing light to the resolution element result in polarimetric signals that are intensity-weighted averages of the contributing atmospheres (Sanchez Almeida, 1997; Leka and Barnes, 2012), which rarely resemble expected ME Stokes spectra (even without complications from unresolved velocity components or gradients within the line-forming region).

The treatment of instrumental scattered light and the approach used to estimate relative contributions of magnetized vs. unmagnetized incoming light (the magnetic “fill fraction” [$f\!\!f$] or percentage of a pixel filled by magnetic field) will influence the inferred nature of magnetic structures, especially (but not solely) unresolved structures (Lites et al., 1996; Socas Navarro, 2004; LaBonte, 2004; del Toro Iniesta, Orozco Suárez, and Bellot Rubio, 2010; Orozco Suárez and Katsukawa, 2012; Leka and Barnes, 2012; Bommier, 2016; Sainz Dalda, 2017). The particulars of how inversion techniques are invoked by which to infer the magnetic field from the spectropolarimetric data, even “standard” Milne–Eddington inversions, will influence the results. The particulars can include the number and which spectral lines are used, plus mundane-seeming choices of optimization algorithms, stopping conditions, and any optimization weighting applied to the $\chi ^{2}$-calculation (see, e.g., Centeno et al., 2014; Sainz Dalda, 2017, for a discussion).

As has been introduced at length by Pevtsov et al. (2021) and Liu et al. (2022), with vector polarimetry and inversions covering the full visible solar disk from, e.g., the *Vector SpectroMagnetograph* (VSM: Keller and The SOLIS Team, 2001; Henney, Keller, and Harvey, 2006) of the *Synoptic Optical Long-term Investigations of the Sun* facility (nso.edu/telescopes/nisp/solis/) and now the *Helioseismic and Magnetic Image* (HMI: Scherrer et al., 2012; Schou et al., 2012; Centeno et al., 2014; Hoeksema et al., 2014) onboard the *Solar Dynamics Observatory* (SDO: Pesnell, Thompson, and Chamberlin, 2012), there is the capability of estimating the vector components of the photospheric magnetic field over large areas of the Sun. Unfortunately, as demonstrated recently (Rudenko and Dmitrienko, 2018; Pevtsov et al., 2021; Sun et al., 2021; Liu et al., 2022), large-scale magnetic structures as observed by these facilities can present behavior that is physically unexpected. Specifically, a preferred direction in polar fields or a “flip” in the direction of the zonal-directed (East/West) horizontal component of magnetic structures is inferred upon crossing the central meridian. Given that the opposite signs of this bias are present in data from different facilities (Pevtsov et al., 2021; Sun et al., 2021), the problem is well-established as originating from instrumentation, data preparation, and/or inversion, rather than being solar in origin. We explore these options further, below.

What has not been emphasized yet is the impact on physical interpretation. The over-estimation of the transverse component $B_{\perp }$ contributes to the inferred physical components $[B_{x}^{h}, B_{y}^{h}, B_{z}^{h}]$, or heliographic / local components of the field vector, in a non-linear way, according to the inherent underlying structure as well the viewing angle [$\theta $] (i.e. the structure’s location on the solar disk).

It has been proposed that the most susceptible features are those that are unresolved. As hypothesized by Pevtsov et al. (2021) and demonstrated by Liu et al. (2022), including the magnetic fill fraction [$f\!\!f$] explicitly as part of a Milne–Eddington solution changes the inferred field vector, such that the bias may be mitigated substantially. This is not a new point (Ronan, Mickey, and Orrall, 1987; Lites and Skumanich, 1990; LaBonte, 2004; Bommier et al., 2007; del Toro Iniesta, Orozco Suárez, and Bellot Rubio, 2010; Sainz Dalda, 2017). Forcing the magnetic fill fraction $f\!\!f = 1.0$ for unresolved structures is likely the dominant source of this bias; as shown by Liu et al. (2022), correcting this assumption by invoking a new version of the Very Fast Inversion of Stokes Vector Milne–Eddington approach (Borrero et al., 2011; Centeno et al., 2014; Griñón-Marín et al., 2021) can mitigate the magnitude of the bias. However, as also shown by Pevtsov et al. (2021), the magnitude of the photon noise can contribute. The imaging spatial integrity, field of view, and acquisition cadence are all drivers of instrument design, the outcomes of which are as different as the questions that they are optimized to answer, cf. SDO/HMI vs. *Hinode*/*SpectroPolarimeter* (SP: Lites et al., 2013), and we use both SDO/HMI and *Hinode*/SP to investigate not just fill-fraction fitting, but noise, instrument, data specifics, and inversion implementation.

Most importantly, we present quantitative metrics by which to evaluate the extent and direction of the bias^1^ and the contributing factors, and finally demonstrate one “quick and dirty” correction that is shown to mitigate (although not necessarily completely correct) the bias in any data for which certain structures are observed and good coverage in observing angle is available.

We begin the methodology section (Section 2) with a description of the data, target acquisition, and analysis methods and evaluation metrics, plus a description of a simple model used in the investigation. This is followed by a summary of results (Section 3) and finally a demonstration of a rudimentary correction approach (Section 4) for when the option of re-inverting the data is not available.

## Methodology

In this section we present the algorithms for selecting features, the observational targets, and analysis methodology.

The overall issue for SDO/HMI pipeline output is that the inferred component in the plane-of-the-sky [$B_{\perp }$ or $B_{\mathrm{trans}}$] is stronger than it “should be” compared to the line-of-sight component [$B_{ \parallel }$ or $B_{\mathrm{los}}$], most apparently for areas with unresolved magnetic structure, i.e. $f\!\!f < 1.0$. For data from the VSM, according to Pevtsov et al. (2021), $B_{\perp }$ it is weaker than it “should be”. We refer to any such imbalance as the bias in $B_{\perp }$ but also refer to the image-plane (as returned from the inversion) inclination $\gamma ^{i}\in [0^{\circ},180^{\circ}]$ where $90^{\circ}$ is in the plane-of-the-sky. In some cases, the “polarity” of $B_{\parallel }$ is inconsequential or adds confusion, in which case, we limit the inclination, referred to as $|\gamma ^{i}| \in [0^{\circ},90^{\circ}]$. Throughout, while we may discuss $f\!\!f$ as distinct from the field magnitude $|B|$, it is their product that is evaluated.

### Target Features

Solar plage is generally agreed to be comprised of kilo-Gauss concentrations of predominantly radially directed field (along the direction of gravity), generally not strong enough to form a continuum depression (but having magnetic and thermal impacts sufficient to produce continuum-intensity enhancements when viewed at an angle), the small size of these concentrations means that they are rarely resolved with today’s instruments. Plage areas are not inherently “weak field” (cf. the “magnetic knots” in Rudenko and Dmitrienko (2018)). The inability to resolve the structures leads to a canonical area-averaged (pixel-averaged) flux-density estimate for plage of $1 - \mathit{few} \times 100$ Mx cm^−2^, depending on instrument specifics. Plage is a wide-spread source for “global-scale” solar magnetic-field structure, and it contributes strongly to synoptic (or synchronic) full-Sun magnetic maps.

The overall approach that we take relies on the analysis of structures on the Sun where the magnetic inclination is known, at least on a statistical basis: plage regions and the darkest umbrae of very stable sunspots. These two targets, when carefully selected, should be dominated by radially directed field (for SDO/HMI spatial resolution, spectral sampling, and sensitivity). It is not required that the orientation be *only* radial, just that it be *dominated* by radially directed field.

In cases where full-disk data are not available, there is an additional assumption invoked, that these structures would display minimal large-scale variation over the course of days, again on a statistical basis (for appropriately stable sunspots).

Different between these two structures are the magnetic fill fraction, with the darkest umbrae of sunspots presenting field-filled pixels ($f \!f = 1.0$), whereas plage should present un-resolved bundles of field within field-free (or field-less) plasma ($f\!\!f < 1.0$). Both plage and sunspots generally present polarization signals well above the noise. The two types of features appropriate for this analysis are selected thus:

A: The very central, darkest portions of sunspot umbrae in spots that are large and not evolving noticeably. To select these areas,
  1. Determine that the size, complexity, morphology, and evolution of the target sunspot are large, nonexistent, round, and steady, respectively.
  2. Create four masks:
    ∘ Mask 1, Continuum intensity: select the darkest points within sunspot umbrae. Specifically, for *Hinode*/SP data: an $I_{\mathrm{c}} / \mathrm{median}(I_{\mathrm{c}})$ limited-FOV image is smoothed with a 4-pixel boxcar, and those pixels $<0.4$ are selected; for SDO/HMI: using $I^{*}$, the normalized continuum intensity from the hmi.Ic_nolimbdark_720s data series (see Hoeksema et al., 2014, for a description of the different data series available from the SDO/HMI pipeline products), choose pixels with $I^{*} < 0.2$. The resulting mask is then dilated by a $2\times 2$ box.
    ∘ Mask 2, Field Strength: $f\!\!f \times B > 200$ Mx cm^−2^; tests showed that this level consistently retains plage areas but does not include noise across orbital variations in SDO/HMI data. This mask is then eroded by a $2\times 2$ box to remove single-pixel detections.
    ∘ Mask 3, Inclination: An image of the “local” or heliographic (or “physical”) $|\gamma ^{h}|$ is created, smoothed using a 4-pixel boxcar, and pixels with $|\gamma ^{h}| < 30^{\circ}$ are identified; this mask is then subjected to first being eroded and then dilated, both using a $2\times 2$ box.
    ∘ Mask 4, Data Quality: for SDO/HMI, mask to include only pixels with conf = 0.0 from the confid_map segment, hmi.ME_720s_fd10 series, that indicate no failures in data quality or inversion (convergence, etc.). For *Hinode*/SP: only those pixels with total polarization $P>0.4$%; those below are used for computing the non-magnetic spectral profiles. For all: within $\mu = \cos (\theta ) > 0.35$ (within $\approx 70^{\circ}$ from disk center).
  3. The four masks are added together and only pixels where all conditions are satisfied are selected.
B: Plage areas are found either in the vicinity of a sunspot, or as active-region remnants that can cover many degrees of the disk. Arguably, any individual plage element evolves, but statistically speaking their distribution is not expected to differ due the hemisphere in which they are located. To select these areas,
  1. Again, construct a series of independent masks:
    ∘ Mask 1, Continuum Intensity: where $I_{\mathrm{c}} / {\mathrm{median}}(I_{\mathrm{c}}) > 0.9$ (*Hinode*/SP) or $I^{*} > 0.9$ (SDO/HMI).
    ∘ Mask 2, Field Strength: as per Mask 2 above, but additionally grown with a $2\times 2$ box to ensure coverage of plage concentrations.
    ∘ Mask 3, Inclination: same as Mask 3, above.
    ∘ Mask 4, Data Quality: same as Mask 4, above.
    ∘ Mask 5, extended sunspot / super-penumbral exclusion: using continuum intensity, select all sunspot points by $I_{\mathrm{c}} / {\mathrm{median}}(I_{\mathrm{c}}) < 0.9$ (*Hinode*/SP) or $I^{*} < 0.9$ (SDO/HMI). The result is eroded using a $2\times 2$ box to remove single-point detections, then grown (dilated) with a large $r=25$-pixel circular template to extend spot areas (including detected pores) beyond the horizontal-field super-penumbra.
  2. Masks 1 – 4 are added together and only pixels where all conditions are satisfied are selected.
  3. Mask 5 provides a negative-Boolean requirement, to remove not-dark but strong fields that can confuse the analysis.

The final masks included an additional identification of the polarity of $B_{z}^{h}$ and the location on the disk (latitude, longitude). Creating the plage masks without Mask 3, meaning without a filter on the local-component inclination angle, results in $\lesssim 5^{\circ}$ difference in, e.g., the mean and medians of the inclination-angle distributions, by including non-radial points. As such, the interpretation of the bias may be systematically incorrect by a small amount, but avoiding having to resolve the $180^{\circ}$ ambiguity may be advantageous in some situations.

### Observational Data

#### *Hinode/SpectroPolarimeter*

**Figure 1:**
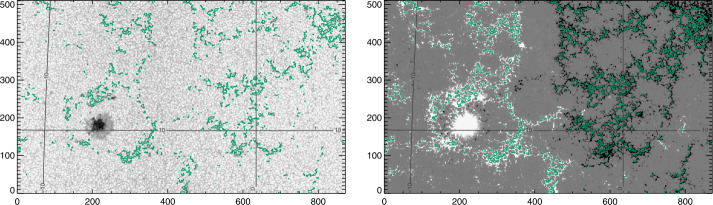
*Hinode*/SP “normal” scan of AR 12457, start time 24 Nov 2015 17:00:05, showing (*left*) continuum (scaled to $1.1\times$median($I_{\mathrm{c}}$)) with latitude/longitude contours, and (*right*) $B_{z}^{h}$, with same latitude/longitude contours. Plage points are identified (green contours). All target-image axes are simply in pixel numbers.

We chose scans from the *Hinode/SpectroPolarimeter* (*Hinode*/SP: Kosugi et al., 2007; Tsuneta et al., 2008; Ichimoto et al., 2008; Lites et al., 2013) that follow NOAA AR 12457 (Table 1). The target includes a small sunspot that does not fit our criteria for spot analysis, but does include a well-developed and minimally evolving plage area (Figure 1). Standard Level-1, the calibrated spectra, and Level-2, the MERLIN Milne–Eddington inversion results, were retrieved; Level 2.1 data were not used here, as we wanted to extend the disambiguation and ensure that it was consistent across inversion experiments (see Section 2.2.3).

**Table 1:** Data / target summary.

Target	Date/Start Time	Location	Notes
Source: *Hinode*/SOT-SP Cadence: 1 day^−1^			
AR 12467	22 Nov 2015 18:50:49	N12 E42	HMI: 22 Nov 2015 19:00:00
^′′^	23 Nov 2015 18:15:05	N12 E29	HMI: 23 Nov 2015 18:24:00
^′′^	24 Nov 2015 17:00:05	N12 E16	HMI: 24 Nov 2015 17:12:00
^′′^	25 Nov 2015 15:45:04	N12 E03	HMI: 25 Nov 2015 16:00:00
^′′^	26 Nov 2015 16:20:04	N12 W10	HMI: 26 Nov 2015 16:36:00
^′′^	27 Nov 2015 15:15:05	N12 W23	HMI: 27 Nov 2015 15:24:00
Source: SDO/HMI Cadence: 96 min.			
Spot 1 [Sp1]	04 Dec 2010 – 12 Dec 2010	N32	NOAA AR 11131
Plage 1 [Pl1]	^′′^	^′′^	Surrounding Spot 1
Plage 3 [Pl3]	^′′^	S23	broad in longitude
Spot 2 [Sp2]	17 May 2016 – 25 May 2016	S07	NOAA AR 12546
Plage 2 [Pl2]	^′′^	^′′^	Surrounding Spot 2
Plage 4 North [Pl4N]	13 May 2016 – 21 May 2016	N07	
Plage 4 South [Pl4S]	^′′^	S06	includes NOAA AR 12547

#### *Solar Dynamics Observatory/Helioseismic and Magnetic Imager*

Data from the *Solar Dynamics Observatory/Helioseismic and Magnetic Imager* (SDO/HMI: Pesnell, Thompson, and Chamberlin, 2012; Scherrer et al., 2012; Schou et al., 2012; Centeno et al., 2014; Hoeksema et al., 2014) provide the known problematic data in this case, plus both time-series and full-disk data for testing inversion options and other mitigating procedures. To match the *Hinode*/SP data of AR 12457, we selected SDO/HMI segments close to the mid-times of the *Hinode*/SP scans (see Table 1) and extracted coincident FOV patches from the full-disk data (*not* as defined by the SDO/HMI Active Region Patch [HARP] 6124’s bounding boxes). The pipeline data included series hmi.ME_720s_fd10 and hmi.B_720s, plus hmi.S_720s for some tests of inversions, and hmi.Ic_nolimbdark_720s for continuum-feature identification.

**Figure 2:**
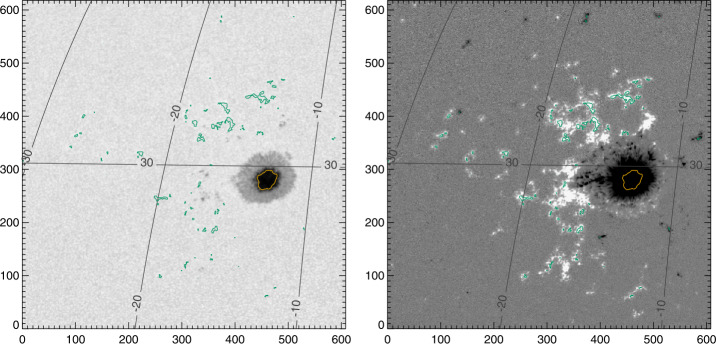
Target sunspot “Spot 1”, NOAA AR 11131, which transited the disk 04 December 2010 – 12 December 2010, at N30, shown here on 07 December 2010 07:24:00 TAI. The surrounding plage is “Plage 1”. *Left*: Continuum, Stonyhurst grid, with axes indicating pixels. *Right*: $B_{z}^{h}$ from the Minimum-Energy disambiguation (see Section 2.2.4), same Stonyhurst grid. The contours indicate selected plage points (*green*) and center-spot radial-field area (*yellow*).

**Figure 3:**
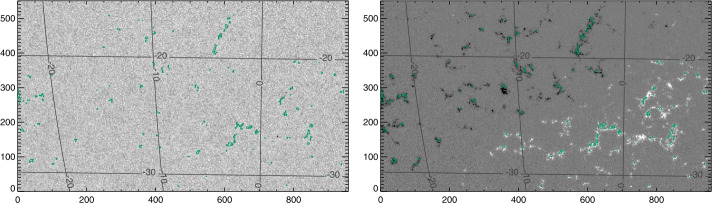
Target “Plage 3”, which transited 04 December 2010 – 12 December 2010 centered at S25, almost directly south of NOAA AR 11131 (see Figure 2), shown here on 07 December 2010 20:12:00 TAI. *Left*: Continuum, Stonyhurst grid, with axes indicating pixels. *Right*: $B_{z}^{h}$ from the Minimum-Energy disambiguation (see Section 2.2.4), same Stonyhurst grid. The contours indicate selected plage points (*green*).

**Figure 4:**
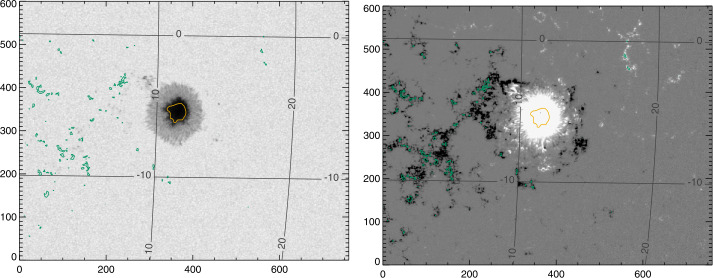
Target “Spot 2”, NOAA AR 12546, and accompanying “Plage 2” , which transited the disk 14 May 2016 – 25 May 2016, shown here on 21 May 2016 06:24:00 TAI. *Left*: Continuum, Stonyhurst grid, with axes indicating pixels. *Right*: $B_{z}^{h}$ from the Minimum-Energy disambiguation (see Section 2.2.4), same Stonyhurst grid. The contours indicate selected plage points (*green*) and center-spot radial-field area (*yellow*).

**Figure 5:**
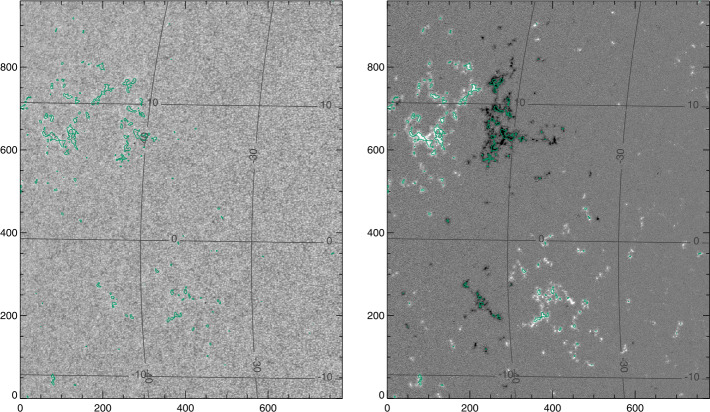
Target plage area “Plage 4” , which transited the disk 13 May 2016 – 21 May 2016, shown on 14 May 2016 08:00:00 TAI. Of note, this region was divided into “Plage 4 North” and “Plage 4 South” for analysis. Plage 4 South eventually produced sunspots and was labeled NOAA AR 12547, but the sunspot points are excluded for plage analysis. *Left*: Continuum, Stonyhurst grid, with axes indicating pixels. *Right*: $B_{z}^{h}$ from the Minimum-Energy disambiguation (see Section 2.2.4), same Stonyhurst grid. The contours indicate selected plage points (*green*).

Two additional time-periods were identified for study, targeting the presence of large, round, stable sunspots and well-distributed plage: 04 December 2010 – 12 December 2010 and 13 May 2016 – 24 May 2016 (Table 1). Not all analysis methods were available for the earlier time-period, but it was valuable to confirm behavior with a second large sunspot and additional plage areas. Custom-sized boxes were defined and extracted at the solar synodic rotation rate, to follow the targeted structures across the solar disk. In addition to the magnetic-field-related segments, we utilized the confidence and the limb-darkened-corrected continuum intensity (as mentioned above). Context images for the targets selected from SDO/HMI time-series data are shown in Figures 2, 3, 4, and 5.

#### Inversion Options

A number of different inversion options were evaluated, comprising two different groups of tests.

The first group used the Milne–Eddington inversion code developed for use with the NCAR/High Altitude Observatory *Advanced Stokes Polarimeter* (labeled ASP-ME; Skumanich and Lites, 1987; Lites and Skumanich, 1990; Leka, 1997) on *Hinode*/SP Level-1 data to systematically evaluate the impact of different implementation options. The options investigated include (see summary in Table 2):

i) optimization scheme: a genetic algorithm or least-squares minimization to obtain the global minimum of $\chi ^{2}$-fit between model atmosphere and observed spectra.
ii) Fitting both 630.15 and 630.25 nm lines or just the 630.25 nm line. Different $g_{\textrm{L}}$-factors imply different sensitivity to $\boldsymbol {B}$ but not to $f\!\!f$, so multiple lines provide additional constraints.
iii) Explicitly fitting for $f\!\!f$ vs. explicitly setting $f\!\!f = 1.0$.
iv) Weights assigned to $S=[I,~Q,~U,~V]$: since the magnitudes of the polarization signals in $[V]$ and [$Q$, $U$] are of order $\mathrm{d} I / \mathrm{d}\lambda $ and $\mathrm{d}^{2} I / \mathrm{d}\lambda ^{2}$, respectively, and photon noise scales accordingly, in order to insure that the $I$ does not dominate the $\chi ^{2}$ in the evaluation functions, weights $[w_{I}, w_{Q} = w_{U}, w_{V}]$ are usually a parameter supplied for the $\chi ^{2}$-calculation. We quote here $w_{{S}}^{\mathrm{eff}} = w_{S} / \sigma _{{S}}$ Care must be taken when comparing codes and their parameter settings, as some request $w_{S}$ while some refer to $w_{S}^{2}$.

The second group compares different inversion codes that may differ in a variety of ways. The different codes are tested on one or both of SDO/HMI hmi.S_720s polarization images or *Hinode*/SP Level-1 spectra, as listed in Table 3. The different systems tested include:

- The pipeline *Hinode*/SP Level-2 output from the MERLIN Milne–Eddington code (heritage from ASP-ME).
- The pipeline output from the Very Fast Inversion of Stokes Vectors (VFISV: Borrero et al., 2011; Centeno et al., 2014) from the hmi.ME_720s_fd10 series.
- The UNNOFit code (Bommier et al., 2007) was applied only to the AR 12457 targets, but with both *Hinode*/SP Level-1 data and SDO/HMI hmi.S_720s data as input. UNNOFit introduced the magnetic filling fraction as a free parameter of the Milne–Eddington inversion. All free parameters describing the non-magnetic part of the atmosphere are set to equal those of the magnetic part, except for the magnetic-field vector itself. This procedure was recently implemented in a new VFISV inversion (see next point), with a slight difference in the series of the eight other parameters, where UNNOFit also determines the Voigt parameter $a$ and eliminates the source function at the photosphere base by normalization. UNNOFit was the first code that explicitly allowed a variable atmosphere for the non-magnetic component, although it is set to be identical to the atmosphere of the magnetic component. However, Bommier et al. (2007) also showed that nine parameters can exceed the information content of the spectra as needed for a successful inversion, and that only the pixel-averaged magnetic-field strength $f\!\!f\times B$ is finally determined.
- A new version of the Very Fast Inversion of Stokes Vector (VFISV_ABGM: Borrero et al., 2011; Griñón-Marín et al., 2021) was developed for implementation with SDO/HMI data to explicitly include a fit for the fill fraction. Similar in the approach to UNNOFit (see above), it should be noted that the Voigt parameter is fixed at $a = 0.5$ and the source function at the base of the photosphere is a fitted parameter. The results from this inversion have been shown to mitigate the bias (Griñón-Marín et al., 2021; Liu et al., 2022). One difference between the VFISV_ABGM data used here and that presented by Griñón-Marín et al. (2021) and Liu et al. (2022) is that we used no polarization threshold for the inversion, whereas a threshold of 0.25% was used in those cited works. We removed the polarization threshold in order to ensure no discontinuity near weak-signal plage areas. No additional scattered-light correction was performed.
- The SyntHIA approach is not really an inversion per se; it is a Convolutional Neural Network that has been trained on the *Hinode*/SP Level-2 output with SDO/HMI hmi.S_720s polarization images as input (Higgins et al., 2021, 2022). The full-disk results provide field and fill-fraction separately, with an overall fidelity closer to *Hinode*/SP pipeline output than SDO/HMI pipeline output.

We additionally test the question of polarization noise. As briefly demonstrated by Pevtsov et al. (2021), noise in the linear polarization $[Q,\,U]$ signals contributes to the bias. We conduct tests comparing inversions using input spectra from the SDO/HMI hmi.S_720s series to those using input spectra from the hmi.S_5760s series, which are 96-minute integrated Stokes spectra, and demonstrably lower in random (photon) noise.

**Table 2:** Tests run on *Hinode*/SP target AR 12457.

Line(s)	*ff* fit	Optimization	$W_{{S}}^{\mathrm{eff}}$	Label	Notes
[nm]	explicitly?	method	*S*=[*I*, *Q*&*U*, *V*]		
Data Source: *Hinode*/SP; Inversion: ASP-ME^a^					
630.15, .25	√	Genetic	[1,10,3.2]	A-DEF	“Default”
630.15, .25	√	Genetic	[1,31.2,3.2]	Aw1	weights-test #1
630.15, .25	√	Least-Squares	[1,31.2,3.2]	ALSw1	
630.15, .25	√	Genetic	[1,10,2.2]	Aw2	weights-test #2
630.15, .25	√	Least-Squares	[1,10,2.2]	ALSw2	
630.15, .25	√	Genetic	[1,1,1]	Aw3	weights-test #3
630.15, .25	√	Genetic	[1,3.2,3.2]	Aw4	weights-test #4
630.15, .25	√	Least-Squares	[1,10,3.2]	ALS	
630.25	√	Genetic	[1,10,3.2]	A1	
630.25	√	Genetic	[1,31.2,3.2]	A1w1	
630.25	√	Genetic	[1,10,2.2]	A1w2	
630.25	√	Least-Squares	[1,10,3.2]	A1LS	
630.25	×	Genetic	[1,10,2.2]	A1NFw2	
630.25	×	Genetic	[1,10,3.2]	A1NF	
630.25	×	Least-Squares	[1,10,3.2]	A1NFLS	
630.15, .25	×	Genetic	[1,10,2.2]	ANFw2	
630.15, .25	×	Genetic	[1,10,3.2]	ANF	
630.15, .25	×	Least-Squares	[1,10,3.2]	ANFLS	
Data Source: *Hinode*/SP; Inversion: OTHER					
630.[15,.25]	√	LM^b^	[0.01,1,0.1]	MERLIN	L2 pipeline^c^
630.15	√	LM	[1,1,1]	UnnoFit_6301	UNNOFit^d^
630.25	√	LM	[1,1,1]	UnnoFit_6302	UNNOFit
Data Source: SDO/HMI; Inversion: VFISV					
617.3	×	LM	[1,3,2]	PIPE	hmi.ME_720s_fd10^e^
617.3	√	LM	[1,3,2]	ABGM	su_abgm^f^
Data Source: SDO/HMI; Inversion: OTHER					
617.3	√	CNN	n/a	SyntHIA	CNN^g^
617.3	√	LM	[1,1,1]	UnnoFit_HMI	UNNOFit

^a^Skumanich and Lites (1987), Leka (1997).

^b^Levenberg–Marquardt (LM) minimization algorithm (see Press et al., 1992).

^c^sot.lmsal.com/data/sot/level2d/.

^d^Bommier et al. (2007).

^e^Centeno et al. (2014).

^f^Griñón-Marín et al. (2021).

^g^Higgins et al. (2021, 2022).

**Table 3:** Tests run on SDO/HMI multi-day time series targets.

*ff* fit?	Optimization	Label	Notes
04 December 2010 05:48:00 – 12 December 2010 04:12:00: Spot 1, Plage 1, Plage 3			
×	LM	PIPE	SDO/HMI pipeline
√	LM	ABGM	su_abgm
√	CNN	SyntHIA	SyntHIA CNN
13 May 2016 20:48:00 – 24 May 2016 19:12:00: Spot 2, Plage 2, Plage 4			
×	LM	PIPE	SDO/HMI pipeline
×	LM	PIPE_5760	Input: hmi.S_5760s
√	LM	ABGM	su_abgm
√	LM	ABGM_5760	Input: hmi.S_5760s, su_abgm
√	CNN	SyntHIA	SyntHIA CNN

Not all possible permutations were executed, but a sufficient number with a sufficient range of options so as to quantitatively judge the impact of the different approaches on the bias. The tests are summarized in Tables 2, 3; please note the method labels will be used in later discussion and figures.

#### Disambiguation

The data from all inversions of both *Hinode*/SP and SDO/HMI data are inherently $180^{\circ}$ ambiguous in the plane-of-the-sky component. All inverted data that were compared using the local components were disambiguated using the “minimum-energy” method (ME0: Metcalf, 1994; Leka, Barnes, and Crouch, 2009; Hoeksema et al., 2014, available at www.nwra.com/AMBIG) with a cooling schedule that generally matched both SDO/HMI and the *Hinode*/SP Level2.1 data products: tfactr = 0.98, neq = 100. The primary difference in how ME0 was called relative to the SDO/HMI pipeline is that lower [athresh, bthres] = [$95,100$] were used to capture more near-plage areas, and the spherical option was deployed for the larger-FOV SDO/HMI data. The local components were then computed using planar or spherical geometry, accordingly.

### Model Data

**Figure 6:**
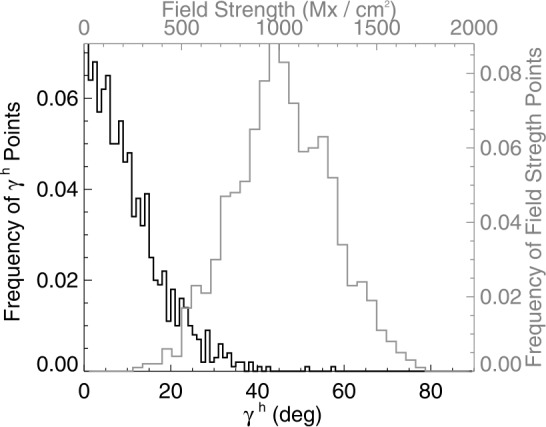
Distributions of heliographic or local inclination angle $\gamma ^{h}$ (*black*) and field strength (*gray*) for the “plage-like” distributions in the model. $\gamma =0$ is radially directed (opposite gravity). These 1000-point samples were then placed across a variety of viewing angles.

To complement the analysis of the observational data, model data were constructed with which to test the hypotheses and the mitigation strategies. On a latitude / longitude grid ranging in both directions $\pm 80^{\circ}$ in $2.5^{\circ}$ increments, 1000-point samples were generated to mimic expected distributions of plage: The radial component is a normally distributed random sample centered at 1000 Mx cm^−2^ with a standard deviation of 250 Mx cm^−2^, the horizontal component was a normal distribution, standard deviation 200 Mx cm^−2^; the azimuthal angle was a uniform distribution across $2\pi $ (Figure 6). Only $B_{z}^{h}> 0$ positive-polarity “plage” was considered here.

From this grid, coordinate transforms re-create the image-plane components $|\boldsymbol {B}|, \gamma ^{i}, \alpha ^{i}$ or alternatively $B_{x}^{i}, B_{y}^{i}, B_{z}^{i}$, the last being $B_{\parallel }$ and $B_{\perp }= \sqrt{{B_{x}^{i}}^{2} + {B_{y}^{i}}^{2}}$. Noise was optionally added at “low”, “medium”, and “high” levels: a normal-distribution random sample with $\sigma = [5, 10, 50]$ Mx cm^−2^ for $B_{\parallel }$ was paired with a normal distribution with $\sigma = [100, 200, 300]$ Mx cm^−2^ for $B_{\perp }$, although the absolute value was added (since the effective photon noise for total linear polarization is positive-definite, see discussion by Pevtsov et al. (2021)). The noise in azimuthal angle was a normal distribution with standard deviation approximately $[2, 4, 6]^{\circ}$. In an attempt to mimic the noise maps of SDO/HMI (see Hoeksema et al., 2014, their Figure 7), we tested adding noise at the levels listed above but scaled as $1.0/\sqrt{\mu}$ such that the noise increases near the limb, but this made little difference in the final outcomes. No attempt was made to model the impact of unresolved field or incorrect fill fraction (although see Leka and Barnes, 2012).

### Analysis and Metrics

Simply detecting a “sign flip” in a re-binned image of a local heliographic field component when a plage area transits the central meridian is a useful initial diagnostic (Pevtsov et al., 2021; Liu et al., 2022). However, it is difficult to quantitatively evaluate the bias in this manner (cf. Rudenko and Dmitrienko, 2018), or determine its full impact.

We develop quantitative metrics based on the assumed physical characteristics of solar structures in order to evaluate the bias. In some cases, temporal sampling is mapped to spatial sampling, again relying on the physical and statistical qualities of plage in that the distributions of the underlying fields are not expected to evolve significantly over the course of a few days.

#### Distributions of $B_{x}^{h}$, $B_{y}^{h}$, $B_{z}^{h}$ with Central Meridian Distance and Observing Angle

**Figure 7:**
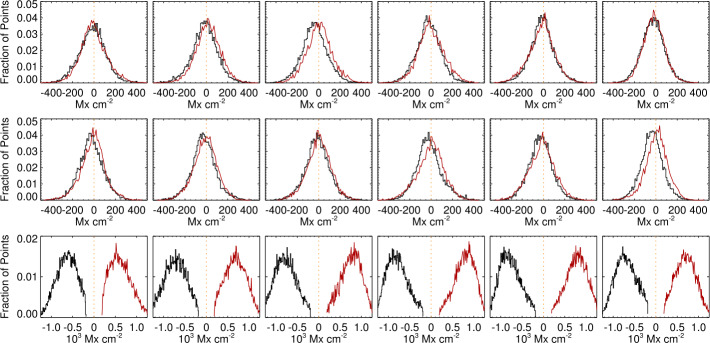
Histograms of plage-identified areas of NOAA AR 12457 for the *Hinode*/SP Level-2 pipeline data output, prepared as described in the text. *Left to Right*: results from the six scans (Table 1); central meridian crossing is at approximately 25 November 2015 00:00, between the third and fourth scans. *Red/Black* indicate pixels that have positive/negative $B_{z}^{h}$; histograms are for $B_{x}^{h}$ (*top*), $B_{y}^{h}$ (*middle*), and $B_{z}^{h}$ (*bottom*). While there is some variation, the distributions for $B_{x}^{h}$ and $B_{y}^{h}$ are essentially independent of polarity and location on the disk.

**Figure 8:**
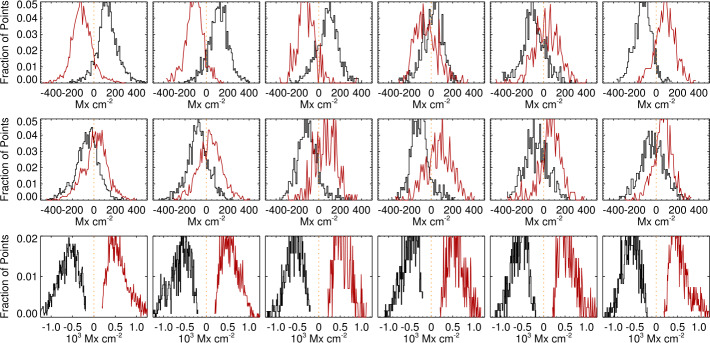
Histograms of plage-identified areas of NOAA AR 12457, and the same field-of-view as in Figure 7, but from SDO/HMI pipeline data, prepared as described in the text. *Left to Right*: results from the six scans (Table 1); central-meridian crossing is at approximately 25 November 2015 00:00, between the third and fourth scans. *Red/Black* indicate pixels that have positive/negative $B_{z}^{h}$; histograms are for $B_{x}^{h}$ (*top*), $B_{y}^{h}$ (*middle*), and $B_{z}^{h}$ (*bottom*). Note the switch of the $B_{x}^{h}$ histogram peaks as the region transits the central meridian, and the sustained but consistent difference in $B_{y}^{h}$.

Histograms of the heliographic $B_{x}^{h}$ are presented separately for pixels with $\pm B_{z}^{h}$, similar to Figure 4 in Pevtsov et al. (2021). Without the presence of filament channels, nearby large active regions, or other phenomena that could introduce a physical direction preference, the assumption is that the histograms should overlap: The distribution of $B_{x}^{h}$ or $B_{y}^{h}$ should not differ according to either the sign of $B_{z}^{h}$ or viewing angle. Tracking the plage in NOAA AR 12457 over six days, we see the very different behavior between the *Hinode*/SP pipeline output (Figure 7) and the SDO/HMI pipeline output (Figure 8), with consistent $<\!B_{x}^{h}\!>$ and $<\!B_{y}^{h}\!>$ regardless of polarity and viewing angle for the former, and a switch of $<\!B_{x}^{h}\!>$ between east/west hemisphere for the latter, as well as a consistent offset in $<\!B_{y}^{h}\!>$.

In situations such as NOAA AR 12457 where there is a sufficient sample of plage with both polarities, the behavior is summarized by metrics describing the differences in the distributions of target magnetic components. Specifically, metrics should target the behavior of the $B_{x}^{h}$ and of $B_{y}^{h}$ most notably, plus $B_{z}^{h}$ when it provides information. The absolute separation is the absolute difference of the medians of two distributions of the target $B_{x}^{h}, B_{y}^{h}$, or $B_{z}^{h}$, the two distributions being whether the underlying field has $B_{z}^{h}\!>\!0$ or $B_{z}^{h}\!<\!0$; the mean absolute separation is taken over the six samples. This is accompanied by the standard deviation of the *signed* separation, which is larger for a switch in sign and smaller if, for example, the distributions are separated but do not change significantly between samples. We additionally consider the median absolute deviation (MAD; ${\mathrm{med}}(|\hat{X} - \tilde{X}|)$, where $\hat{X}$ is the median (most probable) value for the target distribution) and $\tilde{X}$ is its median over the six samples, and the maximum absolute deviation MaxAD, which is the maximum difference between the median of the target distribution (separately for $B_{z}^{h}>0$ or $B_{z}^{h}< 0$) from its expected value: 0.0 for $B_{x}^{h}$ and $B_{y}^{h}$ and the mean $B_{z}^{h}$ over the six samples, as we hypothesize little evolution.

**Figure 9:**
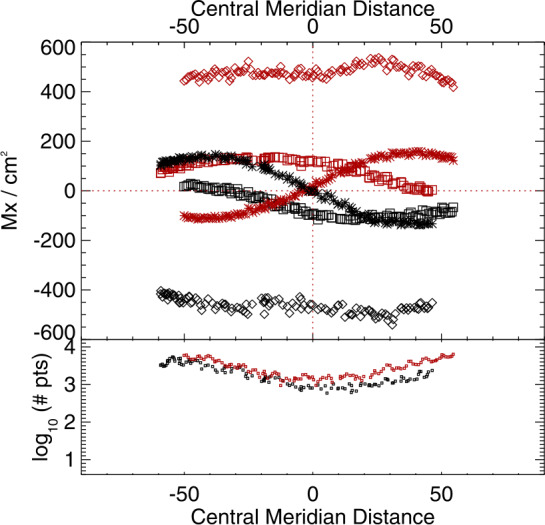
Time series of means of the distributions of $B_{x}^{h}(*), B_{y}^{h}(\Box ), B_{z}^{h}(\Diamond )$ for plage-identified concentrations in “Plage 3” (see Figure 3) and the standard SDO/HMI pipeline (PIPE_720s) output, plus the number of points in the distributions (*lower panel*). *Red/black* indicate positive/negative polarity $B_{z}^{h}$.

**Figure 10:**
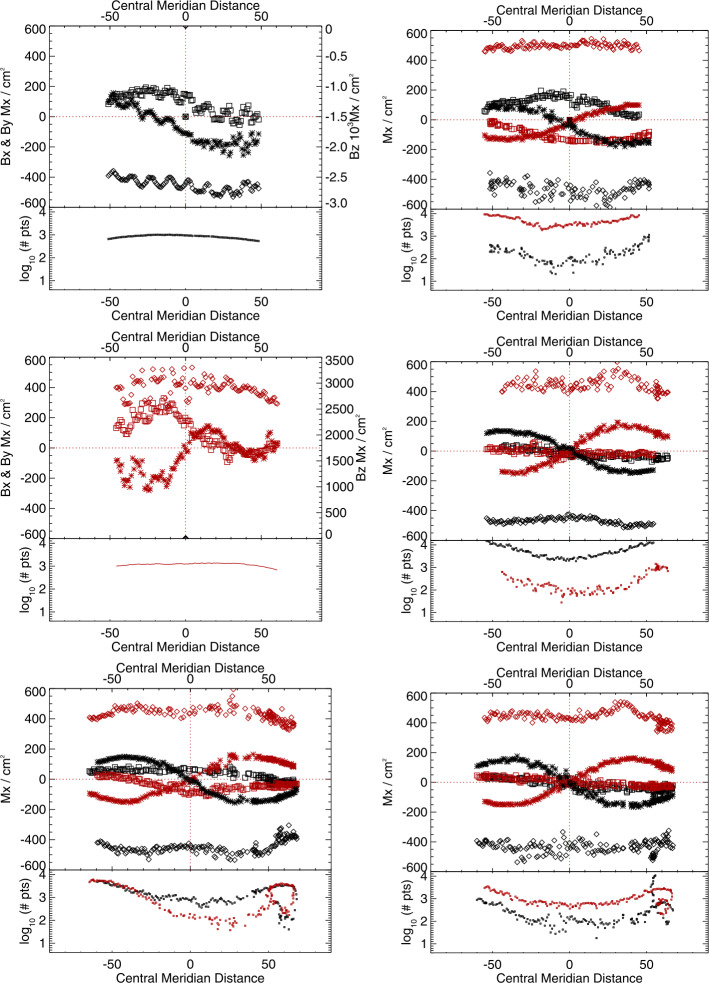
Time series of means of the distributions of $B_{x}^{h}(*), B_{y}^{h}(\Box ), B_{z}^{h}(\Diamond )$. *Red/black* indicate positive/negative polarity $B_{z}^{h}$, cf. Figure 9. (*Top, left/right*): Spot 1, Plage 1, (*Middle, left/right*): Spot 2, Plage 2, (*Bottom, left/right*): Plage 4 North, Plage 4 South. For the spot-related plots, note the different scale (*right-hand* $y$*-axis*) for the $B_{z}^{h}$-component.

This analysis is also applied to the sub-area targeted patches of SDO/HMI time-series data. In Figures 9 and 10 we summarize the behavior of the target distributions as a function of the mid-point longitude for all three vector components. Quantitative summaries take the same form as for the NOAA AR 12457 analysis: mean absolute separation, standard deviation of the signed separation, MAD, and MaxAD.

#### Distributions of $|\gamma ^{i}|$ with Observing Angle

Ideally, there should be metrics available from the image-plane components themselves, without requiring the disambiguation step. If we describe the bias as an over- (or under-) estimate in the $B_{\perp }$ component, then this will manifest in the image-plane inclination angle $\gamma ^{i}$. It is in this context that focusing on plage and the radially oriented locations within stable sunspots becomes useful, because we can assume that for radially directed fields, $B_{r}= B_{\parallel }/ \mu $, where $\mu = \cos (\theta )$ the cosine of the observing angle (Svalgaard, Duvall, and Scherrer, 1978; Wang and Sheeley, 1992). In other words, for radially directed fields without bias, $\gamma ^{i} = \mu $.

**Figure 11:**
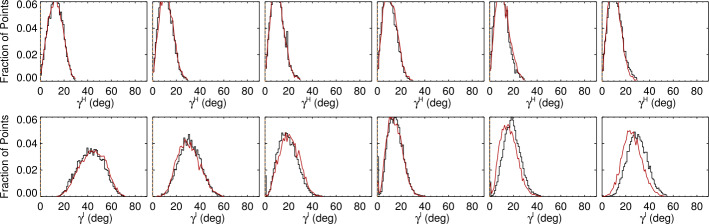
Histograms of plage-identified areas of NOAA AR 12457 for the *Hinode*/SP MERLIN Level-2 pipeline-data output, prepared as described in the text. *Left to Right*: results from the six scans (Table 1); central meridian crossing is at approximately 25 November 2015 00:00, between the third and fourth scans. *Red/Black* indicate histograms of pixels that have positive/negative $B_{z}^{h}$; shown here is the distribution of $\gamma ^{h}$ (*top*) and $\gamma ^{i}$ (*bottom*).

**Figure 12:**
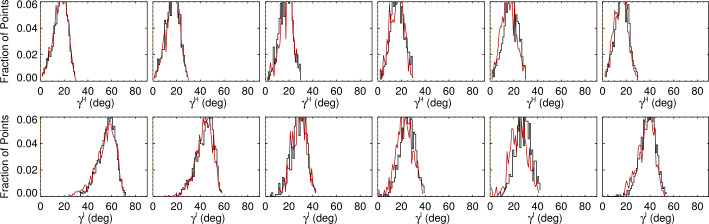
Histograms of plage-identified areas of NOAA AR 12457, and the same field-of-view as in Figure 11, but from SDO/HMI pipeline data, prepared as described in the text. *Left to Right*: results from the six scans (Table 1); central meridian crossing is at approximately 25 November 2015 00:00, between the third and fourth scans. *Red/Black* indicate pixels that have positive/negative $B_{z}^{h}$; shown here is the distribution of $\gamma ^{h}$ (*top*) and $\gamma ^{i}$ (*bottom*), as per Figure 11.

First, we examine the heliographic (local or physical) inclination angle of the target plage points in the NOAA AR 12457 data and confirm that the distributions are the same between the positive and negative regions, change minimally as a function of east/west location, and, while not exactly radial, are not particularly inclined (Figures 11, 12, top panels).

The distribution of $|\gamma ^{i}|$, however, distinctly changes shape as a function of observing angle, and it differs between, e.g., the SDO/HMI and *Hinode*/SP pipeline output (Figures 11, 12, lower panels). The skews of the distributions show exactly opposite behavior with east/west location between the two pipeline data outputs.

**Figure 13:**
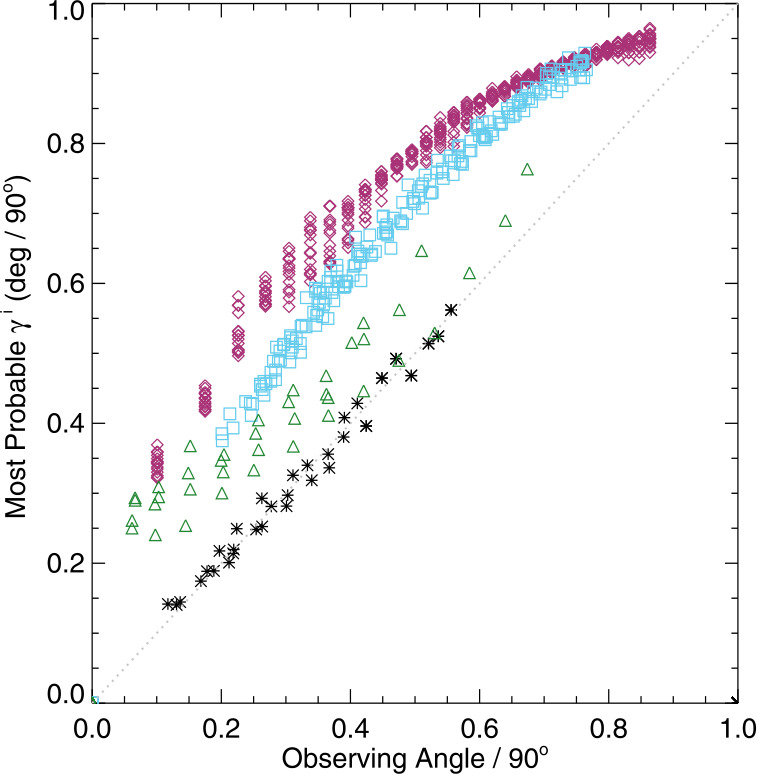
The most probable image-plane absolute inclination $\mathbb{E}(|\gamma ^{i}|)$ ($y$-axis) for plage areas in rings or bins of $\Delta \mu = 0.025$ as a function of the central value for that ring ($x$-axis); both axes are in degrees but normalized by 90^∘^. Shown are *Hinode*/SP MERLIN Level-2.0 data for NOAA AR 12457 for all days sampled (*black* ∗), and three targets from the SDO/HMI VFISV pipeline: full-disk data for 18 May 2016 (*red* $\Diamond $), Plage 3 (*blue* $\Box $), and Spot 2 (*green* △). *Dashed line*: $x=y$. The SDO/HMI full-disk data are sampled for 24 hours at 96 minute cadence (15 samples) and show as well the OBS_VR-related variation (Hoeksema et al., 2014; Schuck et al., 2016).

Figures 11, 12 (top panels) confirm that there is a distribution of orientations within these structures, of course, but they are dominated by radially directed field. Hence we focus on the expected value or most probable value $\mathbb{E}(|\gamma ^{i}|)$, and whether it tracks the observing angle $\mu =\cos (\theta )$ (Figure 13). The degree to which $\mathbb{E}(|\gamma ^{i}|)$ vs. $\theta $ points lie off the $x=y$ line provides information on the bias, including whether there is an under- or over- estimation of $B_{\perp }$. This key diagnostic can be performed on limited-FOV time-series data for (statistically) unchanging structures, but can also be used for full-disk data without requiring any time-series, provided the required structures are present.

In Figure 13, we see that the MERLIN $\mathbb{E}(|\gamma ^{i}|)$ vs. $\theta $ results align with $x=y$ nearly perfectly, while the three examples from the SDO/HMI pipeline all show significant deviations, although with different functional forms. The differences between the two plage-targeted examples could be due to different observing epochs (2010 vs. 2016), or subtle changes in the tracked structures due to evolution or field-of-view. The deviation from $x=y$ for the sunspot confirms the presence of the bias in structures that have strong, pixel-filled signal, presumably with $f\!\!f=1.0$. The different form of $\mathbb{E}(|\gamma ^{i}|)$ vs. $\theta $ for the spot target compared to plage targets may be due to a combination of signal/noise ratio, signal saturation, scattered light treatment, plus the different impact of bias according to the treatment of $f\!\!f$.

The primary metric then to evaluate the distributions of $|\gamma ^{i}|$ for plage as a function of $\mu =\cos (\theta )$ is the Gini coefficient ($\mathcal{G}$) or Receiver (Relative) Operating Characteristic (ROC) Skill Score (ROCSS: Jolliffe and Stephenson, 2012; Leka, Barnes, and Wagner, 2018) $\mathcal{G}=2\times{\mathrm{AUC}} -1$, where the AUC metric is the integrated area under the curve. Provided sufficient coverage is available in $\mu $, $\mathcal{G}$ essentially measures a signed area departure from the $x=y$ line. Other metrics such as the maximum absolute deviation could also be used here, but $\mathcal{G}$ is intuitive, for $\mathcal{G} > 0$ indicates over-estimation of $B_{\perp }$, whereas $\mathcal{G} < 0$ indicates an underestimation. To compute $\mathcal{G}$, we normalize the ranges to $[0,1]$ and extend the sampled ranges to those ends for all data, in order to cursorily account for different sampling in $\mu =\cos (\theta )$ between datasets and tests. The choice of using angles in degrees with a normalization by 90 simply provides an intuitive presentation and AUC calculation; the same information is available using $\mu $ and $\cos (\mathbb{E}(|\gamma ^{i}|))$, similarly both normalized to $[0,1]$.

## Results

There are a large number of tests that were performed; here we summarize the results for targeted questions, first the experiments with model data, then observational.

### The Magnitude and Impact of the Problem

The most obvious impact of this bias manifests in the sign-flip of $B_{x}^{h}$ as extended patches of unresolved structures rotate east/west across the solar disk, as described here and in previous articles (Pevtsov et al., 2021; Liu et al., 2022). The magnitude is demonstrated here quantitatively (Figures 8, 9, 10): up to a ${\mathrm{few}} \times 100~\text{Mx}\,\text{cm}^{-2}$ signal of bias is present, or almost 30% of the total pixel-averaged field magnitude for the plage regions.

It was surmised in those articles that there should be a bias in the north/south distribution of $B_{y}^{h}$ as well. This effect is shown quantitatively here (same figures). The $B_{y}^{h}$-component behaves in an opposite way for plage points with $B_{r}> 0$ vs. $B_{r}< 0$ when located north vs. south of the Equator. The effect is somewhat subtle due to the limited north/south extent available for analysis, but it is clear.

It was also briefly mentioned in previous articles that there may be an impact on the inferred radial component $B_{z}^{h}$ or $B_{r}$ (the component most widely used for global modeling presently), and this is also demonstrated here. It is a smaller effect still, but can be seen as subtle peaks in the most probable pixel-averaged flux away from disk center, and a dip in the magnitude near disk center.

In summary, because this is a problem in the image plane, it impacts all vector components in physical space, including $B_{r}$ ($B_{z}^{h}$). It will also lead to incorrect or biased estimates of any derived quantity such as the vertical current, the force-free parameter $\alpha $, etc. The bias is strongest in *unresolved* structures, and as such will constitute significant portions of the active regions as well as the large-scale plage regions.

It was stated in the earlier articles that there was no bias in the strong-field or $f\!\!f \approx 1.0$ areas. We find this is not actually the case. We show that in the central portions of two large, round, stable sunspots, there is a non-zero most-probable inclination. The most-probable magnetic-field vector changes direction such that $B_{x}^{h}$ changes (or nearly changes) sign as the spots transit the central meridian, and displays a non-zero difference in the most probable value of $B_{y}^{h}$ as well. The variations in $B_{z}^{h}$ are smaller than the orbital-velocity related variations and cannot be confirmed here.

The bias in $f\!\!f \approx 1.0$ areas may be caused ultimately by an unresolved field or the detailed handling of scattered instrumental light (LaBonte, 2004). It indeed fails to cause a full sign reversal of the components, that is true. However, we disagree with Liu et al. (2022) that “This bias does not occur in strong-field regions in sunspots.” The signature of the bias is present, but only apparent with a quantitative examination of the field-component distributions.

### Results from Model Data Experiments

The simple toy model of plage-like distribution of field across a range of observing angles is used to test three possible contributing bias distributions: i) a constant magnitude of bias added to $B_{\perp }$, ii) a contribution to $B_{\perp }$ that is a function of the total field, and iii) a contribution to $B_{\perp }$ that is a function of observing angle, mimicking that which would be expected with systematically higher fill fraction derived in regions away from disk center (hence the bias introduced by an imposed $f\!\!f=1.0$ will be greater toward disk center). The goal here is to reproduce some of the quantitative characteristics observed such as the changing skew of $\gamma ^{i}$-distributions with observing angle and the behavior of the Gini coefficient with bias and its sign, and to understand the source of the varying degree of curvature of $\gamma ^{i}$ as a function of observing angle (Figure 13). In most cases, both a positive and a negative bias are added, meaning bias such as to produce an over-estimate and an under-estimate of $B_{\perp }$, respectively.

**Figure 14:**
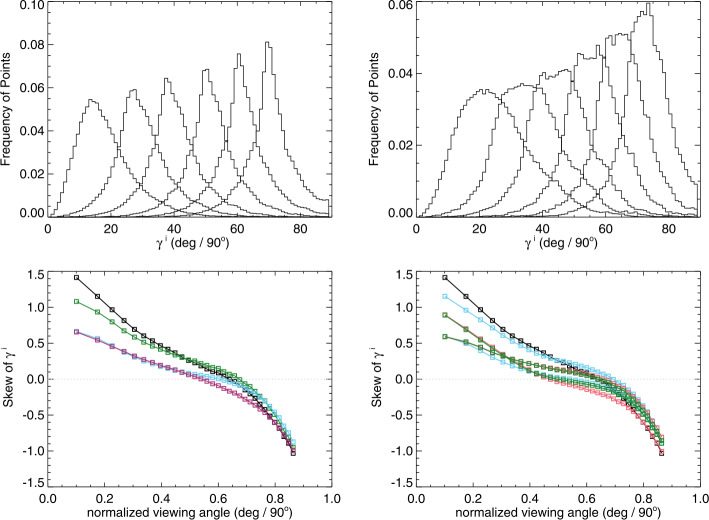
*Top:* histograms of the “observed” $|\gamma ^{i}|$ of the model data for bins of $\Delta\mu=0.025$ centered at $\mu =[0.3625,\,0.50125,\,0.6625,\,0.8125,\,0.9125,\,0.9875]$. (*Left*): “low-noise” case, (*Right*): “high noise” case. *Bottom*: The skew of the $|\gamma ^{i}|$ distributions as a function of viewing angle as the result of different experiments. (*Left*): effect of different levels of photon noise, no additional bias added. *Black*: no noise at all, (*Green*): “low noise”, (*Light Blue*): “medium noise”, and (*Red*): “high noise”. (*Right*): tests of different characterization of bias are shown, as related to the same no noise or bias (*Black*). *Red*: Experiment 1, a constant magnitude of bias at $\pm 200~\text{Mx}\,\text{cm}^{-2}$ is added to $B_{\perp }$; *Light Blue*: Experiment 2, a bias that is $\pm 25$% of the total field strength; *Green*: Experiment 3, a bias of $\pm 200~\text{Mx}\,\text{cm}^{-2} \times \mu $.

The distributions of $|\gamma ^{i}|$ with observing angle show a general trend, as is seen in the *Hinode*/SP data (see Figure 11) of a changing shape with observing angle (Figure 14, Top). The changes are indeed influenced by the degree of photon noise present.

Tracking this change in skew as a function of the experiments performed regarding noise and bias is shown in Figure 14, Bottom. There is a distinct form of the change in skew of the $|\gamma ^{i}|$-distribution as a function of viewing angle, without any additional noise. The addition of photon noise and bias that originates according to the three experiments presents departures from the no-noise case. However, the “optimal” curve (the no-noise no-bias case) is not straightforward to describe: it is not a simple function of observing angle. The curves with added noise and bias depart from the optimal, but in sometimes subtle ways that do not provide unique diagnostic signatures. As such, we elect to not use the skew of the $|\gamma ^{i}|$-distributions as a quantitative test of bias in the observational data.

**Figure 15:**
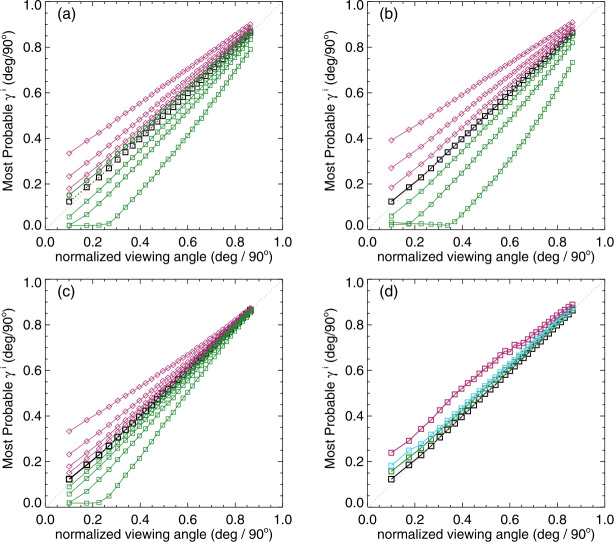
Results of model tests, demonstrating just one approach at a time and its behavior. *Black* is the “no noise, no bias” result for reference; for **a** – **c**, *positive / negative levels are red* $\Diamond $*/green* $\Box $ respectively. (**a**) Experiment 1: no noise, constant $B_{\perp }$ bias at $\pm 50, 100, 200, 400~\text{Mx}\,\text{cm}^{-2}$; (**b**) Experiment 2: no noise, $B_{\perp }$ bias at $\pm 10, 25, 50$% of total pixel-averaged flux; (**c**) Experiment 3: no noise, $B_{\perp }$ bias is added at levels $\pm [50, 100, 200, 400]*\mu $; (**d**) Magnetogram noise only is added (no additional bias) for $B_{\parallel }, B_{\perp }$ respectively at the $[0,0]$ (“no noise” *black*), $[5, 100]$ (“low noise”, *green*), $[10,200]$ (“medium noise”, *light blue*) and $[50,300]$ (“high noise” *red*) levels (see text). For all, the $x=y$ line is indicated.

**Figure 16:**
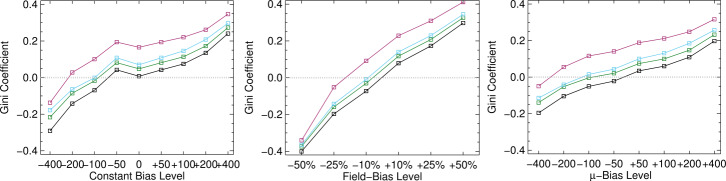
Summary statistics (Gini coefficients $\mathcal{G}$) for the toy-model experiments, here including the behavior of the bias-plus-noise combinations (e.g. adding bias and photon noise, both). *Left*: Experiment 1: “constant Bias Level” applied, *Middle*: Experiment 2: the bias as a fraction of the total field strength, *Right*: Experiment 3: bias level that is then multiplied by $\mu =\cos (\theta )$ for a model of the effect of mis-representing $f\!\!f$. Colors indicate photon noise levels: *Black*: “No Noise”, *Green*: “Low Noise”, *Light Blue*: “Mid Noise”, *Red*: “High Noise”.

The behaviors of the “observed” $\mathbb{E}(|\gamma ^{i}|)$ with viewing angle as per experiments with bias are shown in Figure 15 (Panels a – c) and for photon noise only (Panel d). The “optimal” situation of no photon noise and no bias is the $x=y$ line. Similar to above, the addition of bias produces deviations from the ideal case, but for the bias cases (Panels a – c), the only unique signature is the spread at large viewing angle and a slight difference in shape. To summarize the performance when both bias and noise are included, we present Figure 16, where we focus on the Gini coefficient $\mathcal{G}$ as the bias levels are varied. The black “no noise” cases reflect the curves in Figure 15 a – c, the other curves are as labeled. The impact of including both noise and bias is to increase the departure from $\mathcal{G}=0$ overall and bring even negative bias toward $\mathcal{G}>0$. The latter effect is caused by effectively a “canceling out” effect between the bias and the noise. Given that the noise levels in SDO/HMI data are roughly between the “Mid Noise” and “High Noise” cases, we can possibly use these plots to rule out some extreme cases, but the curves and behaviors are similar enough to probably preclude determining a functional form of the bias.

We find that the impacts of over- or under-estimating $B_{\perp }$ do not always manifest symmetrically about the $x=y$ line for the $\mathbb{E}(|\gamma ^{i}|)$ vs. $\mu $ relation. Furthermore, while the shapes of the curves deviate slightly from linear as both photon noise and bias are added, we are unable with this toy model to replicate the curvature observed in the SDO/HMI pipeline output (Figure 13), even when testing a bias whose behavior is a function of observing angle, as expected by errors in fill fraction. The $\mathbb{E}(|\gamma ^{i}|)$ vs. $\mu $ behavior of Spot 2 is less curved, and closer to the curves produced by the toy model with moderate levels of positive bias. The difference in the $\mathbb{E}(|\gamma ^{i}|)$ vs. $\mu $ behavior (curved vs. closer to linear) between Spot 2 and the plage points in Figure 13 is thus likely due to unresolved structures and hence non-unity $f\!\!f$ in the latter, and how that manifests with observing angle. Further model development to investigate the impact of unresolved structure is beyond the scope of this article (although see the approach by Leka and Barnes, 2012).

### Results from Testing Mitigation Approaches

**Figure 17:**
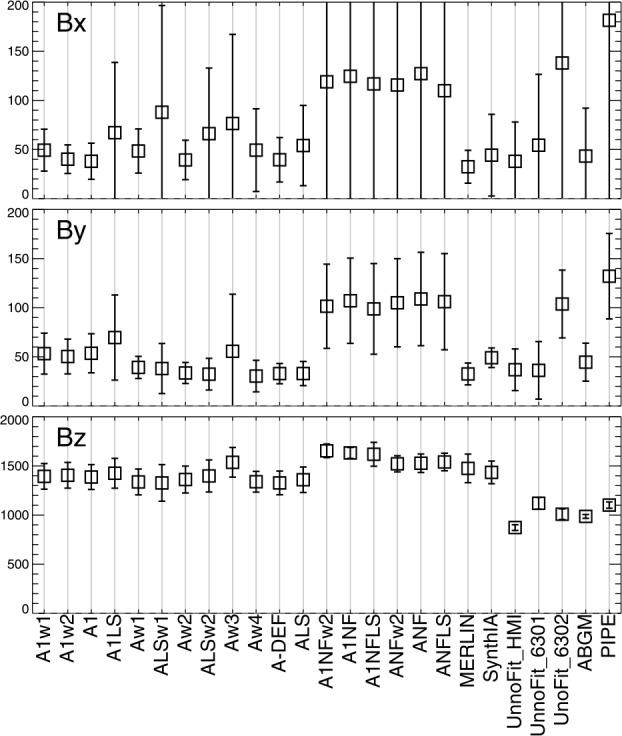
*Boxes*: the mean absolute separation of the most-probable values over the six days for the NOAA AR 12457-based experiments; *error bars*: the standard deviation of the *signed* separation, which will be larger if it changes sign, for example. Results (*top to bottom*) are for $B_{x}^{h}, B_{y}^{h}, B_{z}^{h}$ according to the labels on the $x$-axis (introduced in Table 2).

**Figure 18:**
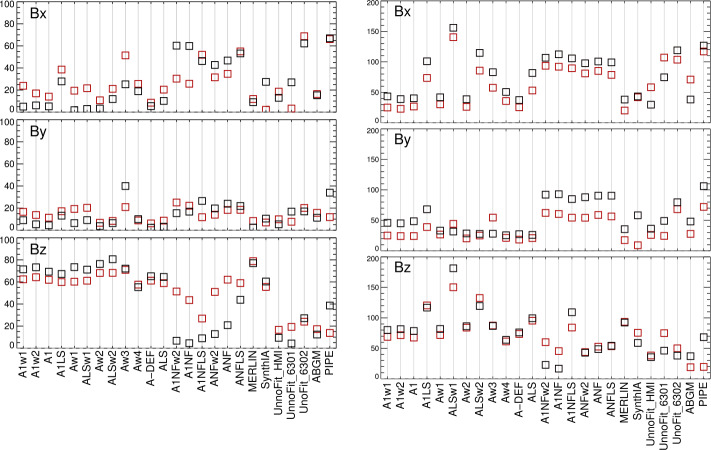
(*Left*): Median absolute deviation (MAD), and (*right*): maximum absolute deviation (MaxAD) of the most probable values over the six samples. *Red/Black* indicate positive / negative underlying $B_{z}^{h}$, respectively.

**Figure 19:**
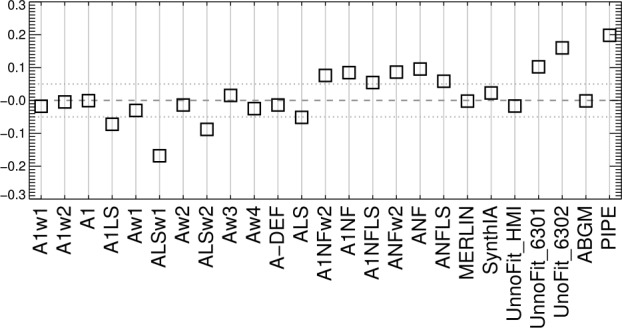
Gini coefficient of the distribution of $|\gamma ^{i}|$ as $f(\mu )$, where the accumulated six maps were combined and then points binned according to $\mu $. Gini coefficients within $\pm 0.05$ should show minimal bias.

**Figure 20:**
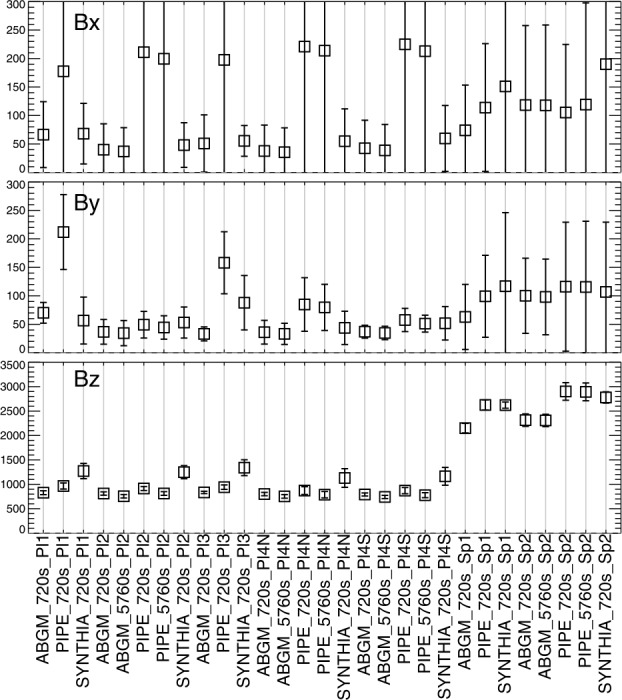
*Boxes*: the mean absolute separation of the most-probable values for the SDO/HMI target patches and inversion options as indicated ($x$-axis labels are introduced in Tables 2, 3, plus the hmi.S_5760s-series integration option), computed for their disk transit; *error bars*: the standard deviation of the *signed* separation, which will be larger if it changes sign, for example. Note that the sunspots are unipolar, which will influence the results.

**Figure 21:**
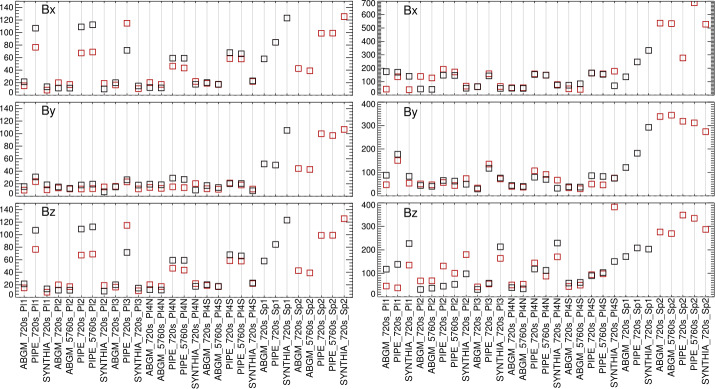
Following Figures 18 and 20, (*Left*) Median absolute deviation (MAD), and (*right*) maximum absolute deviation (MaxAD) of the most probable values over the $\approx 120$ samples as the regions transit the disk. *Red/Black* points are for those with positive / negative underlying $B_{z}^{h}$, respectively; for the spots, there will be only one box for the dominant sign.

**Figure 22:**
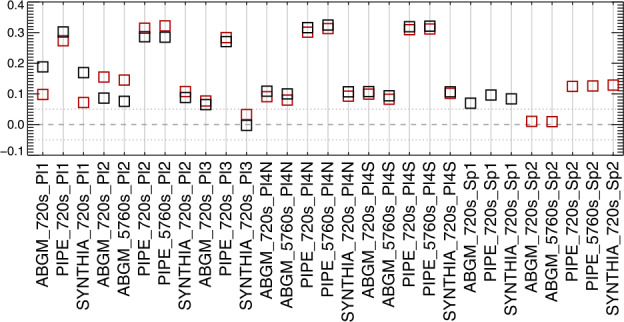
Following Figures 19 and 20, the Gini coefficient of the distribution of $|\gamma ^{i}|$ as $f(\mu )$, as the patches were sampled across the disk. Results within $\pm 0.05$ should show minimal bias.

As discussed in Section 2.2.3, there are other aspects of interpreting specropolarimetric signals that can lead to differences in inferred flux densities. The results of our tests are summarized in Figures 17, 18, and 19, and then in Figures 20, 21, and 22, using the quantitative evaluation metrics on both image-plane $|\gamma ^{i}|$ and heliographic $B_{x}^{h}$-, $B_{y}^{h}$-, and $B_{z}^{h}$-components (see Sections 2.4.1, 2.4.2). While the latter indeed rely on the resolution of the $180^{\circ}$ ambiguity in the $B_{\perp }$ component, the same method was applied throughout, addressing one point made by Sainz Dalda (2017). $\mathcal{G}$ can be positive or negative, all other metrics are positive; all metrics used here tend to zero for best performance (less bias). For all metrics evaluated on time-series data, there may be evolution or field-of-view departures that result in non-zero metrics, but all of the methods are evaluated on consistent data, so comparisons between methods as grouped here are valid.

From the analysis of the AR 12457 data we find that:

∘ Fitting multiple lines vs. only fitting one line in some experiments provided marginal improvement (cf. A1w1 vs. Aw1, A1w2 vs. Aw2) but which line was used and the data treatment also influence the magnitude of the bias (cf. the two UNNOFit results).
∘ MERLIN and ASP-ME default, or A-DEF, results are almost indistinguishable across the metrics, as expected given the heritage of the codes.
∘ The bias is not just a matter of the quality of the input spectropolarimetric data. Inversions of AR 12457 using SDO/HMI input data that account for $f\!\!f$ (SyntHIA, VFISV_ABGM, UNNOFit) were by many metrics almost as good as the MERLIN and ASP-ME (default configuration and others) results using *Hinode*/SP input spectral data.
∘ Optimization method can matter: generally, genetic-algorithm minimization performed slightly better than minimization by least-squares, (cf. Aw1 vs. ALSw1, A-DEF vs. ALS, Aw1 vs. ALSw1), but this is not a strong result in these tests.
∘ Weighting differences can influence the results; equal weighting (Aw3) performed the worst (cf. Aw3 vs. Aw1, Aw2, and Aw4).
∘ Explicitly fitting for the fill fraction provides by far the most significant impact to reduce the bias, (cf. ABGM vs. PIPE, A-DEF vs. ANF, ALS vs. ANFLS).

From the analysis of the SDO/HMI time-series data (of both plage and spots), we find that:

∘ The bias clearly manifests in all three local components of the field, confirming that bias in $B_{\perp }$ contaminates the determination of the true magnetic vector. When the impact of the $B_{\perp }$ bias is high (cf. PIPE_720s_Pl1 vs. ABGM_720s_Pl1 results), it is generally high for all metrics across all three $B_{x}^{h}$-, $B_{y}^{h}$-, $B_{z}^{h}$-components.
∘ The bias can manifest in strong / pixel-filling regions as well as unresolved features. Focusing on *_Sp1 and *_Sp2, while we may expect some evolutionary changes with disk passage, the metrics improve with inversions that fit for $f\!\!f$ (cf. ABGM_720s_Sp1 vs. PIPE_720s_Sp1).
∘ Reducing the photon noise (cf. *720s vs. *5760s) provides a small, but not significant mitigation. Confirming the results of Pevtsov et al. (2021), random photon noise in the data is not the primary source of $B_{\perp }$ bias.
∘ Explicitly accounting for $f\!\!f \neq 1.0$ mitigates the bias, whether through a traditional inversion approach (cf. ABGM_720s_* vs. PIPE_720s_*) or by a neural net trained on inversion output that itself explicitly accounted for $f\!\!f \neq 1.0$ (cf. SYNTHIA_720s_* vs. PIPE_720s_*).

## Post-Facto Quick Fix

Using the assumption that plage is *statistically* dominated by a radially directed field and the fill fraction $f\!\!f$ is not explicitly set or is otherwise already multiplied-through in the equations below, then we use the most probable image-plane inclination $\mathcal{P}(|\gamma ^{i}|) = \theta $, $\theta $ being the observing angle (Svalgaard, Duvall, and Scherrer, 1978; Wang and Sheeley, 1992; Leka, Barnes, and Wagner, 2017), as the basis for evaluation and, now, for correction. This “quick-fix” approach is straightforward (see also Rudenko and Dmitrienko, 2018), but should be implemented when concerned with the large-scale averages or contributions across well-measured (sufficient polarization) pixels. The resulting values are not claimed to be “correct” any more than any inversion results are, just less influenced by the bias.

The approach is as follows for under-resolved plage areas:

i) Identify plage structures sampled across a range of observing angles $\mu =\cos (\theta )$ (with full-disk data or with a temporal sequence of *statistically* consistent structures.
ii) Examine the distributions of $\mathbb{E}(|\gamma ^{i}|)$ as $f(\mu =\cos (\theta ))$ and determine a simple functional form for the systematic angle difference $\Delta |\gamma ^{i}| = \mathbb{E}(|\gamma ^{i}|,\mu ) -\mu $ in appropriate units.
iii) Assuming that $B_{\parallel }$ should not change, and working initially with the absolute inclination $|\gamma ^{i}|$ and assigning a new inclination $|\gamma ^{i}|^{\prime }= |\gamma ^{i}| - \Delta |\gamma ^{i}|$, we find
  1 $$\begin{aligned} B_{\parallel } = & |\boldsymbol {B}| \cos (|\gamma ^{i}|) = |\boldsymbol {B}|^{ \prime }\cos (|\gamma ^{i}|^{\prime}) \end{aligned}$$
  2 $$\begin{aligned} |\boldsymbol {B}|^{\prime } =& |\boldsymbol {B}| \cos (|\gamma ^{i}|^{\prime}) / \cos (|\gamma ^{i}|) \end{aligned}$$
  3 $$\begin{aligned} =& \frac{|\boldsymbol {B}|}{\cos (\Delta |\gamma ^{i}|) + \sin (|\gamma ^{i}|)\sin (\Delta |\gamma ^{i}|)/\cos (\Delta |\gamma ^{i}|)} \end{aligned}$$
  where the $\Delta \gamma ^{i}$ is the functional form of the deviation of the most probable image-frame inclination from the expected inclination as a function of observing angle, and each pixel is thus “corrected” according to field strength, inclination angle, this function, and the observing location. This formulation can of course be presented in a number of ways, but the important aspects are that i) if $\Delta |\gamma ^{i}| \rightarrow 0$ that $|\boldsymbol {B}|$ is recovered, and ii) the results do not become infinite for, e.g., $|\gamma ^{i}|\approx 0$ (if it performs badly near $|\gamma ^{i}|\approx \pi /2$. that will statistically be less of an issue).
  Once the total field strength $|\boldsymbol {B}|^{\prime}$ is adjusted in this way, we work backwards for all points to find their new inclination angle and new components:
  4 $$\begin{aligned} \gamma ^{i\prime} =& \cos ^{-1}(B_{\parallel }/ |\boldsymbol {B}|^{\prime}) \end{aligned}$$
  5 $$\begin{aligned} B_{\perp }^{\prime } =& |\boldsymbol {B}|^{\prime }\sin (\gamma ^{i\prime}) \end{aligned}$$
  where note that in the last two steps, we now use and recover the full $[0,\pi ]$ range of $\gamma ^{i}$. Again, $B_{\parallel }$ remains the same.

**Figure 23:**
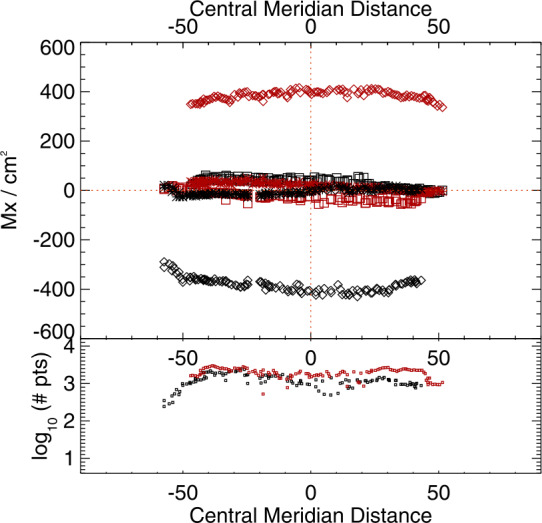
Time series of the means of the distributions of $B_{x}^{h}(*)$, $B_{y}^{h}(\Box )$, $B_{z}^{h}(\Diamond )$ for plage-identified concentrations in “Plage 3” (see Figure 3), but after the corrections described in the text; *lower panel*: number of points in the distributions. *Red/black* indicate positive/negative polarity $B_{z}^{h}$. The metrics, for $B_{x}^{h}$, $B_{y}^{h}$ and $B_{z}^{h}$ respectively are: Mean Absolute Separation: $32.0\pm 25.2$, $57.3\pm 16.6$, $765.6\pm 42.5$; MAD (for $B_{z}^{h}>0\,/\, B_{z}^{h}<0$): $13.5/9.4$, $14.7/12.1$, $12.7/19.0$; MaxAD: $42.4/26.0$, $53.2/57.9$, $42.5/80.4$; Gini: −0.02, −0.02.

As a demonstration, we find the coefficients of a second-order polynomial fit for $\Delta |\gamma ^{i}|$ to $\mu $ from full-disk SDO/HMI data for April 2010, June 2010, and August 2010. A third-order fit the sampled regions well, but extrapolated poorly for disk-center corrections. We then apply the above to the Plage 3 time-series extraction (Figures 3, 9). A first try resulted in an over-correction, diagnosed using a most-probable $|\gamma ^{i}|(\mu )$ plot similar to Figure 13, but a simple 25% reduction in the coefficient magnitudes produced a narrow distribution centered well on the $x=y$ line. The disambiguation was then performed and the same diagnostics are used to evaluate this post-facto approach. The relevant metrics, cf. the results for PIPE_720s_Pl3 in Figures 20 – 22, are listed in the caption for Figure 23 where we show the new time-series plots to compare to Figure 9.

## Concluding Remarks

The interpretation of spectropolarimetric signals in terms of the magnitude, orientation, behavior, and location of magnetic fields in the solar atmosphere is simply not straightforward. How to approach the problem depends severely on the scientific question being posed. With regards to interpreting the large-scale vector field of under-resolved features, it is now well-established that how the spectropolarimetric signals are handled can contribute to a systematic bias in the component perpendicular to the line-of-sight: $B_{\perp }$.

Previous works on this topic have illustrated that the problem exists and touched on some compute-intensive approaches to mitigation. In this work, we perform four novel investigations and point to potentially less-arduous mitigation approaches.

First, we develop quantitative metrics to measure the $B_{\perp }$ bias. At least one, the Gini-coefficient of the departure from expected image-plane inclination angles in plage features, is applicable without performing the $180^{\circ}$ disambiguation, given sufficient sampling of appropriate structures across observing angle. These quantitative metrics are used to point out that the bias in SDO/HMI data is not limited to the unresolved plage features; some bias is seen in the strong-field unity-fill-fraction pixels of sunspots. The quantitative metrics are then available to evaluate and compare data.

Second, we systematically compared the results of different inversion implementation options and targets (different spectral lines used), evaluating the results using common observational targets and the quantitative metrics developed above. We find that the most impactful implementation choice is to include $f\!\!f$ as an independent parameter in the optimization, or having trained a neural net on such data. Applying inversions that explicitly fit $f\!\!f$ mitigates bias in plage *and in strong-field pixel-filled sunspot centers*. That being said, which optimization scheme is used, the weighting used to calculate $\chi ^{2}$, and the use of multiple spectral lines also impact the outcomes. We also tested a direct comparison of two very different data sources (*Hinode*/SP vs. SDO/HMI for NOAA AR 12457), using both pipeline and custom inversions, and found that data source per se did not account for the bias.

Third, we construct a simple “toy” model that is appropriate to test certain observed features of the $B_{\perp }$ bias. Experiments were performed that added bias with different functional forms as well as varying the level of photon noise in the data. We show that noise is not the primary contributor to the of the $B_{\perp }$ bias. We could not, however, reconstruct a distinct non-linear aspect visible in the SDO/HMI $|\gamma ^{i}|(\mu )$ behavior. The source and contribution function to the bias are more complex than our experiments. Further refinement to the models to determine the source of this non-linear shape is beyond the scope of this article.

Fourth and finally, we demonstrate a straightforward “quick fix” that can be applied for analysis of global plage structures on a statistical basis and can be applied prior to performing the disambiguation step. This post-facto algorithm (see also Rudenko and Dmitrienko, 2018), should not take the place of a more robust inversion, and should probably not be used to interpret any individual pixel’s results quantitatively. However, it can be used to produce vector-field data for which the bias is adequately removed so as to produce more reasonable global $B_{\phi }$, $B_{\theta }$, $B_{r}$ maps.

We show that there are in fact viable options for more robust full-disk inversions of SDO/HMI data: UNNOFit (Bommier et al., 2007), the new VFISV_ABGM (Griñón-Marín et al., 2021), and SyntHIA (Higgins et al., 2022). The first two are more traditional implementations of Milne–Eddington codes that explicitly include fill fraction in the optimization, the latter is a neural-net trained on *Hinode*/SP Level-2.0 output and SDO/HMI hmi.s_720s [$I$, $Q$, $U$, $V$] input. Given the vast differential in computing resources required, the latter may be a more readily available solution, especially for large datasets.

## Data Availability

The majority of the data used in this research is sourced from public pipeline-produced products and codes, as cited; specific time-series cubes, for example, can be made available upon request to the corresponding author. Data from VFISV_ABGM are in series su_abgm.ME_720s_fd10_forKD2 available through jsoc2.stanford.edu/ajax/lookdata.html, which requires interested parties to contact the SDO/HMI team for access. Data from SyntHIA can be made available upon reasonable request, see also Higgins et al. (2022) and sources therein; K.D. Leka, E.L. Wagner, and R.E.L. Higgins are actively involved in a project to establish the SyntHIA as a publicly accessible series through JSOC. The UNNOFit inversion code is available at lesia.obspm.fr/perso/veronique-bommier.

## Footnotes

1 The authors became aware of Rudenko and Dmitrienko (2018) effectively after this article was accepted. We want to acknowledge some similarities of our approach to theirs, state that the approaches were developed independently, and direct interested readers to that article for salient points.

## References

[CR1] Bommier V. (2016). Milne-Eddington inversion for unresolved magnetic structures in the quiet Sun photosphere. J. Geophys. Res. Space Phys..

[CR2] Bommier V., Landi Degl’Innocenti E., Landolfi M., Molodij G. (2007). UNNOFIT inversion of spectro-polarimetric maps observed with THEMIS. Astron. Astrophys..

[CR3] Borrero J.M., Tomczyk S., Kubo M., Socas-Navarro H., Schou J., Couvidat S., Bogart R. (2011). VFISV: very fast inversion of the Stokes vector for the helioseismic and magnetic imager. Solar Phys..

[CR4] Centeno R., Schou J., Hayashi K., Norton A., Hoeksema J.T., Liu Y., Leka K.D., Barnes G. (2014). The Helioseismic and Magnetic Imager (HMI) vector magnetic field pipeline: optimization of the spectral line inversion code. Solar Phys..

[CR5] del Toro Iniesta J.C., Orozco Suárez D., Bellot Rubio L.R. (2010). On spectropolarimetric measurements with visible lines. Astrophys. J..

[CR6] del Toro Iniesta J.C., Ruiz Cobo B. (2016). Inversion of the radiative transfer equation for polarized light. Liv. Rev. Solar Phys..

[CR7] Griñón-Marín A.B., Pastor Yabar A., Liu Y., Hoeksema J.T., Norton A. (2021). Improvement of the Helioseismic and Magnetic Imager (HMI) vector magnetic field inversion code. Astrophys. J..

[CR8] Henney C.J., Keller C.U., Harvey J.W., Casini R., Lites B.W. (2006). SOLIS-VSM solar vector magnetograms. Solar Polarization 4.

[CR9] Higgins R.E.L., Fouhey D.F., Zhang D., Antiochos S.K., Barnes G., Hoeksema J.T., Leka K.D., Liu Y., Schuck P.W., Gombosi T.I. (2021). Fast and accurate emulation of the SDO/HMI Stokes inversion with uncertainty quantification. Astrophys. J..

[CR10] Higgins R.E.L., Fouhey D.F., Antiochos S.K., Barnes G., Cheung M.C.M., Hoeksema J.T., Leka K.D., Liu Y., Schuck P.W., Gombosi T.I. (2022). SynthIA: a synthetic inversion approximation for the Stokes vector fusing SDO and Hinode into a virtual observatory. Astrophys. J. Suppl..

[CR11] Hoeksema J.T., Liu Y., Hayashi K., Sun X., Schou J., Couvidat S., Norton A., Bobra M., Centeno R., Leka K.D., Barnes G., Turmon M. (2014). The Helioseismic and Magnetic Imager (HMI) vector magnetic field pipeline: overview and performance. Solar Phys..

[CR12] Ichimoto K., Lites B., Elmore D., Suematsu Y., Tsuneta S., Katsukawa Y., Shimizu T., Shine R., Tarbell T., Title A., Kiyohara J., Shinoda K., Card G., Lecinski A., Streander K., Nakagiri M., Miyashita M., Noguchi M., Hoffmann C., Cruz T. (2008). Polarization calibration of the solar optical telescope onboard Hinode. Solar Phys..

[CR13] Jolliffe I.T., Stephenson D.B. (2012). Forecast Verification: A Practioner’s Guide in Atmospheric Science.

[CR14] Keller C.U., Sigwarth M., The SOLIS Team (2001). The SOLIS Vector-Spectromagnetograph (VSM). Advanced Solar Polarimetry – Theory, Observation, and Instrumentation.

[CR15] Kosugi T., Matsuzaki K., Sakao T., Shimizu T., Sone Y., Tachikawa S., Hashimoto T., Minesugi K., Ohnishi A., Yamada T., Tsuneta S., Hara H., Ichimoto K., Suematsu Y., Shimojo M., Watanabe T., Shimada S., Davis J.M., Hill L.D., Owens J.K., Title A.M., Culhane J.L., Harra L.K., Doschek G.A., Golub L. (2007). The Hinode (Solar-B) mission: an overview. Solar Phys..

[CR16] LaBonte B. (2004). The imaging vector magnetograph at Haleakala: III. Effects of instrumental scattered light on Stokes spectra. Solar Phys..

[CR17] Leka K.D. (1997). The vector magnetic fields and thermodynamics of sunspot light bridges: the case for field-free disruptions in sunspots. Astrophys. J..

[CR18] Leka K.D., Barnes G. (2012). Modeling and interpreting the effects of spatial resolution on solar magnetic field maps. Solar Phys..

[CR19] Leka K.D., Barnes G., Crouch A., Lites B., Cheung M., Magara T., Mariska J., Reeves K. (2009). An automated ambiguity-resolution code for Hinode/SP vector magnetic field data. The Second Hinode Science Meeting: Beyond Discovery-Toward Understanding.

[CR20] Leka K.D., Barnes G., Wagner E.L. (2017). Evaluating (and improving) estimates of the solar radial magnetic field component from line-of-sight magnetograms. Solar Phys..

[CR21] Leka K.D., Barnes G., Wagner E.L. (2018). The NWRA classification infrastructure: description and extension to the discriminant analysis flare forecasting system (DAFFS). J. Space Weather Space Clim..

[CR22] Lites B.W., Skumanich A. (1990). Stokes profile analysis and vector magnetic fields. V – The magnetic field structure of large sunspots observed with Stokes II. Astrophys. J..

[CR23] Lites B.W., Leka K.D., Skumanich A., Martinez Pillet V., Shimizu T. (1996). Small-scale horizontal magnetic fields in the solar photosphere. Astrophys. J..

[CR24] Lites B.W., Akin D.L., Card G., Cruz T., Duncan D.W., Edwards C.G., Elmore D.F., Hoffmann C., Katsukawa Y., Katz N., Kubo M., Ichimoto K., Shimizu T., Shine R.A., Streander K.V., Suematsu A., Tarbell T.D., Title A.M., Tsuneta S. (2013). The Hinode spectro-polarimeter. Solar Phys..

[CR25] Liu Y., Griñón-Marín A.B., Hoeksema J.T., Norton A.A., Sun X. (2022). On the hemispheric bias seen in vector magnetic field data. Solar Phys..

[CR26] Metcalf T.R. (1994). Resolving the 180-degree ambiguity in vector magnetic field measurements: the ‘minimum’ energy solution. Solar Phys..

[CR27] Orozco Suárez D., Katsukawa Y. (2012). On the distribution of quiet-sun magnetic fields at different heliocentric angles. Astrophys. J..

[CR28] Pesnell W.D., Thompson B.J., Chamberlin P.C. (2012). The Solar Dynamics Observatory (SDO). Solar Phys..

[CR29] Pevtsov A.A., Liu Y., Virtanen I., Bertello L., Mursula K., Leka K.D., Hughes A.L.H. (2021). On a limitation of Zeeman polarimetry and imperfect instrumentation in representing solar magnetic fields with weaker polarization signal. J. Space Weather Space Clim..

[CR30] Press W.H., Teukolsky S.A., Vetterling W.T., Flannery B.P. (1992). Numerical Recipes: The Art of Scientific Computing.

[CR31] Ronan R.S., Mickey D.L., Orrall F.Q. (1987). The derivation of vector magnetic fields from Stokes profiles: integral vs. least squares fitting techniques. Solar Phys..

[CR32] Rudenko G., Dmitrienko I. (2018). The presence of a systematic error in SDO/HMI data. J. Solar-Terr. Phys..

[CR33] Sainz Dalda A. (2017). A statistical comparison between photospheric vector magnetograms obtained by SDO/HMI and Hinode/SP. Astrophys. J..

[CR34] Sanchez Almeida J. (1997). Physical properties of the solar magnetic photosphere under the MISMA hypothesis. I. Description of the inversion procedure. Astrophys. J..

[CR35] Scherrer P.H., Schou J., Bush R.I., Kosovichev A.G., Bogart R.S., Hoeksema J.T., Liu Y., Duvall T.L., Zhao J., Title A.M., Schrijver C.J., Tarbell T.D., Tomczyk S. (2012). The Helioseismic and Magnetic Imager (HMI) investigation for the Solar Dynamics Observatory (SDO). Solar Phys..

[CR36] Schou J., Scherrer P.H., Bush R.I., Wachter R., Couvidat S., Rabello-Soares M.C., Bogart R.S., Hoeksema J.T., Liu Y., Duvall T.L., Akin D.J., Allard B.A., Miles J.W., Rairden R., Shine R.A., Tarbell T.D., Title A.M., Wolfson C.J., Elmore D.F., Norton A.A., Tomczyk S. (2012). Design and ground calibration of the Helioseismic and Magnetic Imager (HMI) instrument on the Solar Dynamics Observatory (SDO). Solar Phys..

[CR37] Schuck P.W., Antiochos S.K., Leka K.D., Barnes G. (2016). Achieving consistent Doppler measurements from SDO/HMI vector field inversions. Astrophys. J..

[CR38] Skumanich A., Lites B.W. (1987). Stokes profile analysis and vector magnetic fields. I – Inversion of photospheric lines. Astrophys. J..

[CR39] Socas Navarro H. (2004). Multiline Stokes analysis for the study of small-scale solar magnetic fields. Astrophys. J..

[CR40] Sun X., Liu Y., Milić I., Griñón-Marín A.B. (2021). Are the magnetic fields radial in the solar polar region?. Res. Notes Am. Astron. Soc..

[CR41] Svalgaard L., Duvall T.L., Scherrer P.H. (1978). The strength of the Sun’s polar fields. Solar Phys..

[CR42] Tsuneta S., Ichimoto K., Katsukawa Y., Nagata S., Otsubo M., Shimizu T., Suematsu Y., Nakagiri M., Noguchi M., Tarbell T., Title A., Shine R., Rosenberg W., Hoffmann C., Jurcevich B., Kushner G., Levay M., Lites B., Elmore D., Matsushita T., Kawaguchi N., Saito H., Mikami I., Hill L.D., Owens J.K. (2008). The solar optical telescope for the Hinode mission: an overview. Solar Phys..

[CR43] Wang Y., Sheeley J.N. (1992). On potential field models of the solar corona. Astrophys. J..

[CR44] Westendorp Plaza C., del Toro Iniesta J.C., Ruiz Cobo B., Martinez Pillet V., Lites B.W., Skumanich A. (1998). Optical tomography of a sunspot. I. Comparison between two inversion techniques. Astrophys. J..

[CR45] Wright, P.: 2017, *ColourBlind: A Collection of Colour-blind-friendly Colour Tables*, Zenodo. DOI. ADS.

